# Supplementary material for: Understanding recruitment to a perioperative randomised controlled trial: protocol for a mixed-methods substudy nested within a feasibility trial of octreotide infusion during liver transplantation
Source: BMJ Open. 2022 Jul 19;12(7):e060177. doi: 10.1136/bmjopen-2021-060177 (PMC9301788; doi:10.1136/bmjopen-2021-060177)
Supplement: Supplementary data [file bmjopen-2021-060177supp001.pdf]

Appendices

Appendix 1: Study flowchart from RCT protocol (submitted for publication; manuscript ID: bmjopen-2021-055864.R1).

Provisional consent will be obtained while patient is on the waiting list. At admission confirmation of consent will be followed by enrolment, randomisation and treatment.

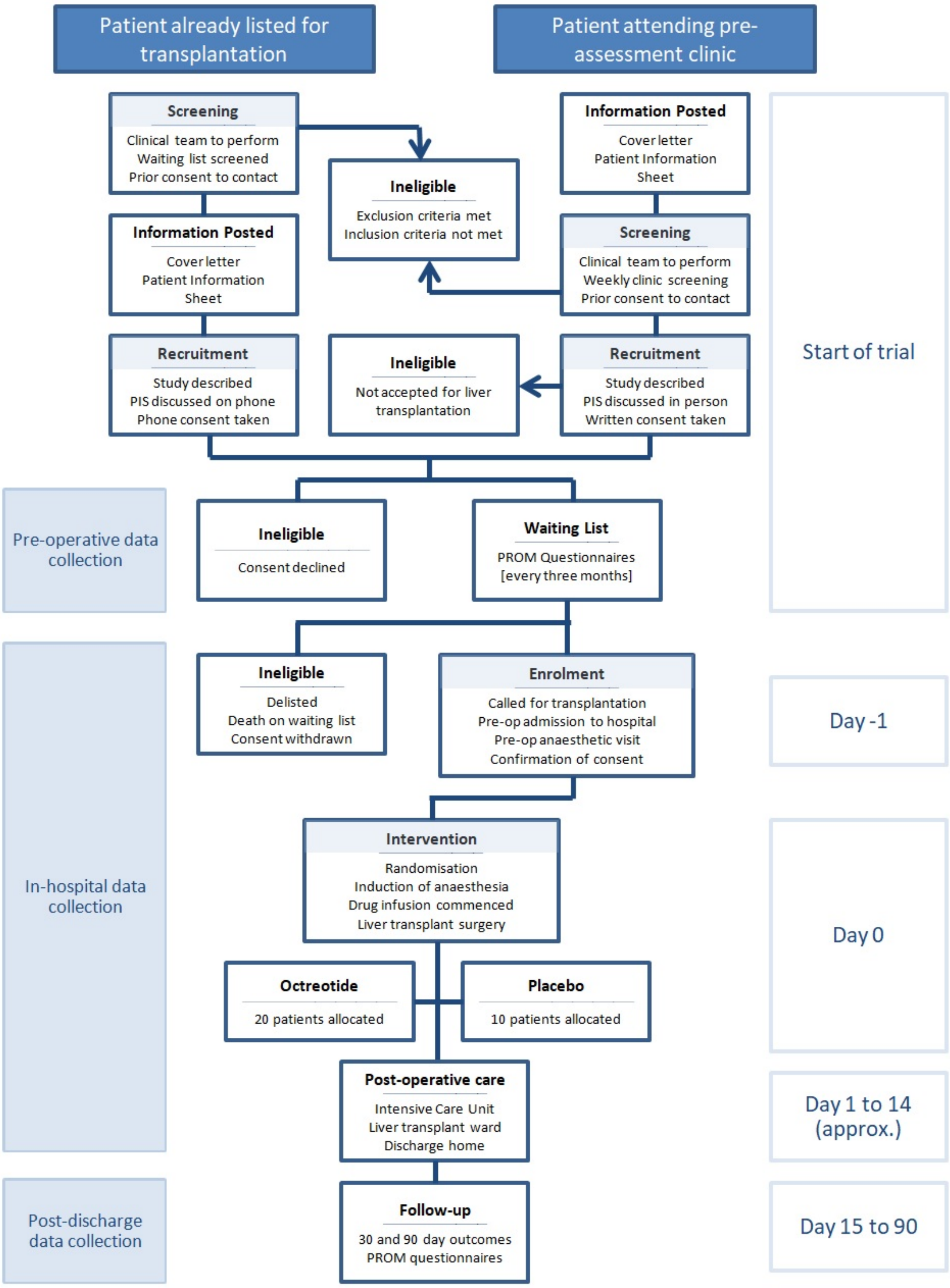

Appendix 2: Participant Information Sheet – for patients

Understanding patient recruitment to a perioperative RCT. A qualitative study.

Participant Information Sheet (Patients)

Introduction

We would like to invite you to participate in this sub-study exploring recruitment to a randomised controlled trial, 'Assessing the Impact of Octreotide Infusion during Liver Transplantation' (henceforth referred to as 'the Trial'). We are interested in learning about why patients are willing (or not) to participate in the Trial. To do this, we will invite all patients who are approached to participate in the Trial to complete a questionnaire. The questionnaire will explore views on the Trial and reasons for agreeing or declining to participate. If recruitment rates to the Trial drop below predetermined levels, or if the Trial team have concerns about the recruitment process, we will seek permission from patients to record recruitment consultations and interview them afterwards by telephone. Recorded interviews will be transcribed (written up) and the tape will then be wiped clean. We will also interview members of the Trial team. Any contributions you make will be anonymised and collated as part of a larger group.

Why is this study being done?

We hope to understand and improve processes for recruiting patients to the Trial.

What will happen if I complete a Questionnaire?

After discussing the Trial with the study team (your 'recruitment consultation'), you will be invited to complete and return a questionnaire. The questionnaire will explore your views on the Trial and reasons for agreeing or declining to participate. All information gathered will be treated as confidential by the study personnel and anonymised for analysis.

What will happen if I take part in the recording of recruitment consultations and subsequent Interviews?

If you are asked, and consent for us to do so, we will use a digital voice recorder during your recruitment consultation. We will use this recording to analyse how the Trial was discussed with you. At least 24 hours later, we will conduct a telephone interview with you to ask you about your views of the Trial, the recruitment process, and your reasons for accepting or declining to participate. The interview will be recorded. All information gathered will be treated as confidential by the study personnel and anonymised for analysis. We will use our findings to help the Trial team improve their recruitment processes.

How long will the study last?

The total duration of the study will be two years but your involvement will only be for the questionnaire you submit, or for the consultation recording and subsequent interview.

Can I stop being in the study?

You can decide to stop participating at any time. After a questionnaire, recruitment consultation or interview has been concluded, you are free to decline consent to any future involvement in the study but historic data will not be destroyed.

What risks can I expect from being in the study?

This is a very low risk study. Information you provide about your experiences and opinions will be documented, but your name will not be used in any reports of the information provided. The information obtained from these observations will only be used by the project researchers and will be locked at our project offices. Hard copies of research data will be shredded after 10 years, and securely disposed of in confidential waste. We will do our best to make sure that any personal information gathered for this research study is kept private and treated in accordance with the Data Protection Act 2018 (https://www.gov.uk/government/collections/data-protection-act-2018).

Are there benefits to taking part in the study?

There will be no direct benefit to you from participating in this study. However, the information that you provide will help the Trial team and future researchers understand and improve recruitment to surgical and perioperative trials which we hope will benefit future groups of patients.

What other choices do I have if I do not take part in this study?

You are free to choose not to participate in the study. If you decide not to take part in this study, there will be no penalty to you, it will not affect your clinical care or your participation in the Trial.

What are the costs of taking part in this study? Will I be paid for taking part in this study?

There are no costs to you for taking part in this study. You will not be paid for taking part in this study.

What are my rights if I take part in this research study?

Taking part in this study is your choice. You may choose either to take part or not to take part in the study. No matter what decision you take, there will be no penalty to you in any way.

Who can answer my questions about the research study?

You can talk to the researchers about any questions or concerns you have about this study. Their contact details can be found below. If for any reason you do not wish to do this, please see the section below.

Giving consent to participate in the research study

You may keep this information sheet if you wish. Participation in this study is voluntary. You have the right to decline to participate in the study without penalty. If you do not wish to participate, you should inform the researcher when given a questionnaire, or at the beginning of the recruitment consultation/interview. If you do

not agree to quotes or other results arising from your participation in the study being included, even anonymously, in reports about the study, please tell the researcher.

Who is organising and funding the research study?

The study is organised by the Royal College of Anaesthetists and led by Prof SR Moonesinghe, a Consultant in Intensive Care Medicine and Anaesthesia at UCLH, and Professor of Anaesthesia and Perioperative Medicine at UCL. Prof Moonesinghe is assisted by a multidisciplinary project team consisting of anaesthetists, surgeons and patients. The research costs for the study have been supported by a grant from the National Institute for Health Research.

Who has reviewed the research study?

The study design has been reviewed by the UCL Research Ethics Committee and the Health Research Authority before any patients or staff were approached to participate.

What will happen to the results?

The results will be analysed and used to help improve processes for recruiting patients to the Trial. In addition, results will be written up for publication in scientific journals and for presentation at conferences. All information gathered will be anonymised and will not be traceable to you.

"What if there is a problem" or "What happens if something goes wrong?"

If you wish to complain, or have any concerns about any aspect of the way you have been approached or treated by members of staff you may have experienced due to your participation in the research, please contact the research team. UCL complaints mechanisms may also be available to you, or the Patient Advice and Liaison Service (PALS) at your hospital (see below).

Chief Investigator: Prof SR Moonesinghe **email:** ramani.moonesinghe@nhs.net

Researcher: Dr Duncan Wagstaff **email:Duncan.wagstaff@nhs.net**

UCL Centre for Perioperative Medicine, Charles Bell House, 43-45 Foley Street, London, W1W 7TS

Appendix 3: Participant Information Sheet – for recruiters

Understanding patient recruitment to a perioperative RCT. A qualitative study.

Participant Information Sheet (Recruiters)

Introduction

We would like to invite you to participate in this sub-study exploring recruitment to a randomised controlled trial, 'Assessing the Impact of Octreotide Infusion during Liver Transplantation' (henceforth referred to as 'the Trial'). We are interested in learning about why patients are willing (or not) to participate in the Trial. To do this, we are surveying and interviewing patients, recording recruitment consultations, and also interviewing members of the Trial team. Any contributions you make will be anonymised and collated as part of a larger group.

Why is this sub-study being done?

We hope to understand and improve processes for recruiting patients to the Trial.

What will happen if I take part in the interviews?

You may be sent an email and subsequently approached by a researcher to invite you take part in an interview. This participant information sheet will be provided in advance of you being interviewed, and with sufficient time (at least 24 hours) to be able to ask any questions you may have. Having had your questions answered satisfactorily, you will be asked to sign a consent form at the start of the interview. The interview will be conducted in private and last approximately 30-60minutes during which time we will ask you some questions about the Trial, its recruitment processes and how you think they might be improved. We will take notes of the discussion and an audio recording will also be made using a digital voice recorder. Recorded interviews will be transcribed (written up) and the tape will then be wiped clean. All information gathered will be treated as confidential by the study personnel and anonymised for analysis.

How long will the sub-study last?

The total duration of the study will be two years but your involvement will only be for your interview.

Can I stop being in the sub-study?

You can decide to stop participating at any time. After an interview has been concluded, you are free to decline consent to any future involvement in the study but historic data will not be destroyed.

What risks can I expect from being in the sub-study?

This is a very low risk study. Information you provide about your experiences and opinions will be documented, but your name will not be recorded or used in any reports of the information provided. The information obtained from these interviews will only be used by the project researchers and will be locked at our project offices. Hard copies of research data will be shredded after 10 years, and securely disposed of in confidential

waste. We will do our best to make sure that any personal information gathered for this research study is kept private and treated in accordance with the Data Protection Act 2018 (https://www.gov.uk/government/collections/data-protection-act-2018).

Are there benefits to taking part in the sub-study?

There will be no direct benefit to you from participating in this study. However, the information that you provide will help the Trial team and future researchers understand and improve recruitment to surgical and perioperative trials.

What other choices do I have if I do not take part in this sub-study?

You are free to choose not to participate in the study. If you decide not to take part in this study, there will be no penalty to you.

What are the costs of taking part in this study? Will I be paid for taking part in this sub-study?

There are no costs to you for taking part in this study. You will not be paid for taking part in this study.

What are my rights if I take part in this research sub-study?

Taking part in this study is your choice. You may choose either to take part or not to take part in the study. No matter what decision you take, there will be no penalty to you in any way.

Who can answer my questions about the sub-study?

You can talk to the researchers about any questions or concerns you have about this study. Their contact details can be found below. If for any reason you do not wish to do this, please see the section below.

Giving consent to participate in the sub-study

You may keep this information sheet if you wish. Participation in this study is voluntary. You have the right to decline to participate in the study without penalty. If you do not wish to participate, you should inform the researcher when given a questionnaire, or at the beginning of the recruitment consultation/interview. If you do not agree to quotes or other results arising from your participation in the study being included, even anonymously, in reports about the study, please tell the researcher.

Who is organising and funding the sub-study?

The study is by Prof SR Moonesinghe, a Consultant in Intensive Care Medicine and Anaesthesia at UCLH, and Professor of Anaesthesia and Perioperative Medicine at UCL. Prof Moonesinghe is assisted by a multidisciplinary project team consisting of anaesthetists, surgeons and patients. The research costs for the study have been supported by a grant from the National Institute for Health Research.

Who has reviewed the sub-study?

The sub-study has been reviewed by the UCL Research Ethics Committee and the Health Research Authority before any patients or staff were approached to participate.

What will happen to the results?

The results will be analysed and written up for publication in scientific journals and for presentation at conferences. All information gathered will be anonymised and will not be traceable to you.

"What if there is a problem" or "What happens if something goes wrong?"

If you wish to complain, or have any concerns about any aspect of the way you have been approached or treated by members of staff you may have experienced due to your participation in the research, please contact the research team. UCL complaints mechanisms may also be available to you.

Chief Investigator: Prof SR Moonesinghe **email:** ramani.moonesinghe@nhs.net

Researcher: Dr Duncan Wagstaff **email:Duncan.wagstaff@nhs.net**

[illegible]

Appendix 4: Consent Form

CONSENT FORM Participant Identification Number:

Title of Project: **Understanding Patient Recruitment to a Perioperative RCT**

Chief Investigator: **Dr Ramani Moonesinghe**

Please initial box

I confirm that I have read and understand the Participant Information Sheet (version 1.2) for the above study. I have had the opportunity to consider the information, ask questions and have had these answered satisfactorily ☐

I understand that my participation in an interview or recording of recruitment consultation is voluntary and that I am free to withdraw at any time without giving any reason, without my employment or legal rights being affected. ☐

I understand that data and quotations I provide may be used (anonymised fully) in future publications of this research. ☐

I understand that audio recordings of the interviews and/or recruitment consultations will be made and stored anonymously. The recording will be deleted once it has been written up. ☐

☐

I understand that, in the event of my withdrawal from the study, data I provide prior to my withdrawal will be retained (anonymised fully) for analysis and publication.

I understand that data collected during the study may be looked at by individuals from University College London (UCL) or from regulatory authorities where it is relevant to my taking part in this research. I give permission for these individuals to have access to my records. Information will be treated as strictly confidential and handled in accordance with the provisions of the General Data Protection Regulation. ☐

☐

I agree to take part in the above study.

____________________ ____________ _______________

Name of Participant Date Signature

____________________ ____________ _______________

Name of Person Date Signature

taking consent

When completed: 1 for participant; 1 (original) for researcher site file

Appendix 5: Patient Questionnaire

Clinical Trials Questionnaire

We are interested in the reasons why patients accept or decline to take park in the clinical trial of octreotide infusion during liver transplantation. We would be grateful if you could complete and this questionnaire. It will not be shown to your doctor or any of the staff at the hospital.

	Yes	No	Not decided yet
Did you agree to take part in the trial mentioned above?			

Below are some reasons that may have influenced your decision to accept or decline to take part in this trial. Please answer each question by ticking the box that shows most clearly how you feel.

	Strongly agree	Agree to some extent	Unsure	Disagree to some extent	Strongly disagree
1) I thought the trial/study offered the best treatment available.					
2) I believed the benefits of treatment in the trial/study would out-weigh any side-effects.					
3) I was satisfied that either treatment in the trial/study would be suitable for me.					
4) I was worried that my illness would get worse unless I joined the trial/study.					
5) The idea of randomisation worried me.					

6) I wanted the doctor to choose my treatment rather than be randomised by computer					
7) The doctor told me what I needed to know about the trial					
8) I trusted the doctor treating me.					
9) I was given too much information to read about the trial.					
10) I was given enough information to read about the trial.					
11) I knew that I could leave the trial at any time and still be treated.					
12) I did not feel able to say no.					
13) I wanted to help with the doctors' research.					
14) I feel that others will benefit from the results of the trial.					
15) The doctor wanted me to join the trial.					
16) Others (e.g. family/friends) wanted me to join the trial.					

Which as the most important reason for you out of this list? (Please give number)_______________

Are there any other reasons for your decision? Please list them below

Thank you very much for completing this questionnaire.

Appendix 6: Interview Topic Guide – for patients

Understanding Patient Recruitment to a Perioperative RCT.

INTERVIEW TOPIC GUIDE

1. Did you agree to take part in the study?
2. What were your reasons for agreeing/declining to take part?
3. How did you feel about the written information you received before speaking to the study team?
4. After speaking to the study team, did you understand everything that you wanted to about the trial?
5. What did you understand the potential benefits of participation might be?
6. What did you understand the potential harms of participation might be?
7. Was there any other information that you wanted to know before making your decision?
8. How did you feel about the process of randomisation?
9. Did discussions with family/friends affect your reasons for agreeing/declining to take part in the trial, and if so, in which ways?
10. Did your doctor(s) want you to take part in the trial?
11. Is there anything else that you'd like to mention?

Appendix 7: Interview Topic Guide – for recruiters

Understanding Patient Recruitment to a Perioperative RCT.

INTERVIEW TOPIC GUIDE - recruiters

1. Please describe your role in the study

2. How do you feel about the **written information** about the trial which is provided for participants before they are recruited? (i.e. timing, format, content)

3. How do you feel about the processes for recruiting patients?
    a. Location
    b. Timings
    c. Information sheets
    d. Consent form

4. What do you think potential participants understand the potential **benefits** of participation might be?

5. What do you think potential participants understand the potential **risks** of participation might be?

6. What are the main reasons you think that patients **agree** to take part?

7. What are the main reasons you think that patients **decline** to take part?

8. Are there any aspects of the trial which are particularly hard to explain to participants?

9. How do you think patients feel about the process of randomisation?

10. Do you think discussions with family/friends affect patients' decisions for agreeing/declining to take part in the trial, and if so, in which ways?

11. How do you think the recruitment process(es) could be improved?

12. Is there anything else that you'd like to mention?
