# Supplementary material for: Understanding recruitment to a perioperative randomised controlled trial: protocol for a mixed-methods substudy nested within a feasibility trial of octreotide infusion during liver transplantation
Source: BMJ Open. 2022 Jul 19;12(7):e060177. doi: 10.1136/bmjopen-2021-060177 (PMC9301788; doi:10.1136/bmjopen-2021-060177)
Supplement: Author's manuscript [file bmjopen-2021-060177.draft_revisions.pdf]

BMJ Open

BMJ Open is committed to open peer review. As part of this commitment we make the peer review history of every article we publish publicly available.

When an article is published we post the peer reviewers' comments and the authors' responses online. We also post the versions of the paper that were used during peer review. These are the versions that the peer review comments apply to.

The versions of the paper that follow are the versions that were submitted during the peer review process. They are not the versions of record or the final published versions. They should not be cited or distributed as the published version of this manuscript.

BMJ Open is an open access journal and the full, final, typeset and author-corrected version of record of the manuscript is available on our site with no access controls, subscription charges or pay-per-view fees (http://bmjopen.bmj.com).

If you have any questions on BMJ Open's open peer review process please email info.bmjopen@bmj.com

BMJ Open

Understanding recruitment to a perioperative randomised controlled trial: protocol for a mixed-methods sub-study nested within a feasibility trial of octreotide infusion during liver transplantation

Journal:	*BMJ Open*
Manuscript ID	bmjopen-2021-060177
Article Type:	Protocol
Date Submitted by the Author:	15-Dec-2021
Complete List of Authors:	Brodkin, Edgar; Royal Free London NHS Foundation Trust, Department of Anaesthesia<br>Loh, Ee-Neng; Royal Free London NHS Foundation Trust, Department of Anaesthesia<br>Spiro, Michael; Royal Free London NHS Foundation Trust, Department of Anaesthesia; University College London, Department of Surgical Biotechnology, Division of Surgery and Interventional Science<br>Hannon, Vivienne; Royal Free London NHS Foundation Trust, Department of Anaesthesia<br>Fabes, Jez; Peninsula Medical School; Royal Free London NHS Foundation Trust, Department of Anaesthesia<br>Moonesinghe, S. Ramani; University College London, Centre for Perioperative Medicine, Research Department of Targeted Intervention<br>Wagstaff, Duncan; University College London, Centre for Perioperative Medicine, Research Department of Targeted Intervention
Keywords:	QUALITATIVE RESEARCH, STATISTICS & RESEARCH METHODS, Transplant surgery < SURGERY, Clinical trials < THERAPEUTICS, TRANSPLANT SURGERY

SCHOLARONE™
Manuscripts

Understanding recruitment to a perioperative randomised controlled trial: protocol for a mixed-methods sub-study nested within a feasibility trial of octreotide infusion during liver transplantation

E Brodkin[1], EN Loh[1], M Spiro[1,2], V Hannon[1], J Fabes[1,3], SR Moonesinghe[4], D Wagstaff[4]

[1] Department of Anaesthesia, Royal Free London NHS Foundation Trust, London, United Kingdom
[2]Department of Surgical Biotechnology, Division of Surgery and Interventional Science, University College London, United Kingdom
[3] Peninsula Medical School, University of Plymouth, Plymouth, United Kingdom
[4]Centre for Perioperative Medicine, Research Department of Targeted Intervention, Division of Surgery and Interventional Science, University College London, London, United Kingdom

Email addresses of authors:

E Brodkin – edgarbrodkin@nhs.net
EN Loh - ee-neng.loh@nhs.net
M Spiro - michaelspiro@nhs.net
V Hannon - vivienne.hannon@nhs.net
J Fabes - jeremy.fabes@nhs.net
SR Moonesinghe - ramani.moonesinghe@nhs.net
D Wagstaff - duncan_wagstaff@yahoo.co.uk

Correspondence to: Dr Duncan Wagstaff, email duncan_wagstaff@yahoo.co.uk, mobile number +447812125650.
Centre for Perioperative Medicine, Department for Targeted Intervention, Division of Surgery and Interventional Science, University College London, Charles Bell House, 43-47 Foley St, London W1T 7AA UK.

Word count (excluding title page, abstract, references, footnotes, figures and tables): 1813

For peer review only

Abstract

Introduction Recruitment to perioperative randomised controlled trials is known to be challenging. Qualitative methods offer insight into barriers and enablers to participation. This is a sub-study within a feasibility randomised controlled trial of octreotide infusion during liver transplantation at two NHS hospitals, which will evaluate patient and staff experiences of trial processes. By sharing formative understanding from these methods with the trials team we aim to improve staff-patient interactions and hence recruitment rates.

Methods and Analysis This prospective mixed-methods study will comprise two workstreams. First, after consent to the randomised controlled trial is sought, all patients will be invited to complete a questionnaire to explore their perceptions of the information given to them and motivating factors that influenced their decision to consent or not. Questionnaires will be analysed using descriptive statistics and Framework Analysis.

If the recruitment:approach ratio drops below a predetermined ratio or if there are any specific recruitment concerns from the trials team, a second workstream involving mixed-methods fieldwork will be implemented. This will involve audio-recording of recruitment consultations and a follow-up semi-structured interview to explore patients' perception of their decision-making regarding recruitment. Semi-structured interviews will also be conducted with the recruitment team to establish their views about the trial, barriers to recruitment and ways to overcome them. Recruitment consultations will be analysed using Q-QAT methodology and interviews will be analysed using Framework Analysis. Findings from both workstreams will be formatively fed back to the trials team to enable iterative improvement to recruitment processes.

Ethics and Dissemination Approval has been granted by Greater Manchester West Research Ethics Committee (ref 20/NW/0071), the Health Research Authority and the local Research and Development offices. A manuscript detailing the summative findings will be submitted to peer-reviewed journals.

Registration The study has been registered with the Study Within a Trial database (ref 152).

Key Words Qualitative research, statistics and research methods, transplant surgery, clinical trials.

For peer review only

Article Summary

Strengths and limitations of this study

- The use of qualitative methodologies will give us in-depth insight into the barriers and enablers to recruitment to this perioperative randomised controlled trial (RCT)
- Data gathered across two sites will make the findings more generalisable to other centres
- Formative feedback to the RCT team during ongoing recruitment will allow modification of the recruitment process to improve recruitment rates
- The role of the two of the researchers (EB, DW) as anaesthetists and members of the RCT trial management group (TMG) may affect the conduct of the recruitment team interviews

For peer review only

Introduction

Multicentre, double-blind RCTs are the methodology of choice for investigating the effectiveness and safety of healthcare interventions.[1] Previous research has shown that recruitment to RCTs can be challenging.[2] Barriers to participation may include, for example, concern over the concept of randomisation, incomplete explanations of trial methodology, or a lack of balance in the way that treatment arms are explained to potential participants by research staff.[2] These difficulties in recruitment can result in failing to start, abandoning or revising target sample sizes of RCTs.[3–5] Furthermore, in multicentre trials, poor or unequal recruitment can negatively impact on staff morale, equality of workload and trial costs in addition to introducing bias and reducing statistical power.[6]

Previous systematic reviews have shown that methods implemented to improve recruitment to research studies in general have not shown clear benefit. Multicentre trials are also under-represented in this literature.[7] However, Donovan et al.[6,8] have demonstrated how contemporaneous qualitative research methods can improve the rates of randomisation and informed consent in multicentre RCTs, including in the perioperative setting. They described how with improved understanding of recruitment processes (both 'as planned', and 'as done'), as well as patient perceptions of recruitment, can be formatively fed back to trial teams to enable timely adjustments to be made.

This mixed-methods sub-study is nested within a double-blind randomised, placebo-controlled feasibility trial investigating the use of octreotide infusion during liver transplantation at two NHS hospitals. Octreotide infusions are currently used during liver transplantation in some centres to potentially reduce bleeding, improve renal outcomes and improve haemodynamic status. However, this practice is based on observational studies. This trial (henceforth referred to as 'the RCT') will be the first to assess the feasibility of randomising patients undergoing liver transplantation to receive either octreotide infusion or placebo. The protocol for the RCT has already been submitted for publication (Manuscript ID: bmjopen-2021-055864.R1).

Consent for participation and recruitment into the RCT will be sought when patients come for an inpatient assessment prior to being placed on the transplant waiting list. Patients already on the waiting list will be contacted by telephone. As there can be a prolonged time period on the transplant waiting list, consent will be confirmed ('enrolment') on admission to hospital for their transplant (Appendix 1). This strategy has been successfully implemented in comparable interventional studies in this population.[9] The focus of our study will be the initial recruitment stage.

For peer review only

Aims & Objectives

Aim

To evaluate the barriers and enablers to recruitment to the RCT.

Objectives

1. To survey patients' reasons and motivation for participation in the RCT using a written questionnaire
2. In the event of low recruitment rates, to undertake in-depth mixed-methods evaluation of consultation recordings and patient/staff interviews with regards to the recruitment processes
3. To provide formative and summative feedback to the RCT team, which will enable necessary adjustments to the recruitment process

Research Questions

1. What are the barriers and enablers to patient recruitment to this RCT?
2. Can the recruitment processes be optimised to improve recruitment throughout the feasibility study or for any follow-on substantive trials?

For peer review only

Methods and Analysis

Study Design

This is a nested mixed-methods sub-study of a feasibility RCT exploring the use of an octreotide infusion during orthotopic liver transplantation across two centres; the Royal Free Hospital (RFH), and University Hospital Birmingham (UHB). The RFH will be the lead site where the team who led the grant application and study design are based.

This sub-study comprises two workstreams: a questionnaire of all patients approached for recruitment to the RCT; and mixed-methods fieldwork. The mixed-methods fieldwork will only be undertaken if the observed recruitment:approach ratio is below predetermined thresholds of <0.3 at the RFH, or < 0.15 at University Hospitals Birmingham (UHB), after at least 12 patients at each site, or if the RCT team have specific concerns about recruitment processes. The predetermined thresholds have been chosen to reflect recruitment rates during a previous RCT in this patient population at the RFH,[9] and lower recruitment rates at secondary centres.

Interim findings from the sub-study will be fed back to the Trial Management Group (TMG) of the RCT at fortnightly meetings. This will enable us to pick up and respond to themes we identify both during the feasibility study and potentially in our planned full randomised trial and will enable the TMG to make timely adjustments to recruitment processes where needed. Summative findings will be provided to the various stakeholders including the patient representative and the clinical teams.

Patient and public involvement

The patient representative of the TMG, who has experience with both liver transplantation and recruitment to research studies, was involved in reviewing and refining study design and methodology. Their feedback was used to draft and amend study documentation. They will continue to be involved for the duration of the study and will have input into the dissemination of both the formative and summative findings.

Eligibility and consent

All patients eligible for the RCT will be eligible for recruitment to the sub-study. Completion of the questionnaire will be taken as implied consent. Participants who are approached for recruitment to the RCT, during their inpatient assessment or via telephone, will be provided with a written patient information sheet (PIS) together with the contact details of the recruitment team (Appendix 2). They will be given at least 24 hours to review the PIS prior to their recruitment consultation. The sub-study will be discussed with them (either in person

For peer review only

during the admission for inpatient assessment or by telephone) prior to the recruitment consultation.

If the mixed-methods fieldwork is initiated, all members of the recruitment team and all patients approached for trial participation will be eligible for recruitment. Individuals will only be excluded if they refuse to provide written consent or if they do not speak English. The recruitment team will also be given at least 24 hours to review the PIS prior to their interviews (Appendix 3).

Recruitment

All patients approached for consent to the feasibility RCT will be invited to complete the questionnaire after that consultation. The RCT aims to *enrol* 30 patients within 10 months but will likely have to *recruit* a far greater number of patients to achieve this as not all patients on the liver transplant waiting list will receive a graft organ within the recruitment timeframe (Appendix 1). There is therefore no specific targeted sample size for the questionnaires; this phase will conclude when recruitment to the RCT ends.

If the mixed-methods fieldwork workstream is triggered, subsequent consecutive patients will be asked for consent (Appendix 4) to have their recruitment consultation (face to face or telephone) recorded and then be interviewed afterwards. Recruitment will continue until theoretical saturation has been achieved; this is likely to occur after approximately 12 patients have been recruited. Recruiters at both sites will be interviewed until theoretical saturation has been achieved (likely to be 6 recruiters).

Data Collection

The questionnaire is based on a previously validated questionnaire used in a similar scenario.[10] It explores patients' perceptions of the trial information given to them and their rationale behind agreeing or declining to consent for the RCT. This has been adapted for our study with input from a patient expert on the TMG (Appendix 5). For the patients contacted via telephone, questionnaires will be completed in hard copy and returned to the study team.

The fieldwork will comprise three different approaches: audio recordings of the recruitment consultation, patient interviews and recruiter interviews.

Audio recordings of face-to-face and telephone recruitment consultations to the RCT will be taken via an encrypted digital voice recorder. Semi-structured interviews with patients will be conducted by telephone at least 24 hours after the recruitment consultation. This will be recorded using an encrypted digital voice recorder. The interviews will focus on the patients'

For peer review only

perception of the information they were given about the recruitment consultation and the rationale behind their decision to consent to the RCT or not.

Semi-structured interviews with members of the research team will be conducted in person or via telephone and recorded on an encrypted digital voice recorder and professionally transcribed.

All interviews will be performed by experienced interviewers who are not members of the trial TMG and do not undertake trial recruitment. All recordings will be professionally transcribed. Interview Topic Guides (Appendices 6 and 7) will be used to ensure consistency and will be updated iteratively based on feedback from the TMG.

Data Analysis

Questionnaires will be analysed formatively every 20 patients approached and summatively at the end of the feasibility study. Responses to closed questions will be summarised using descriptive statistics and thematic analysis for any open-ended answers.

Audio recordings of recruitment consultations will be analysed according to the Q-QAT methodology:[11] this involves summarising recruitment consultations both quantitatively and qualitatively. The quantitative component records the time taken to present each of the RCT treatments (mean, median range; recruiter; centre), the time taken to explain the design, purpose and procedures of the RCT (mean, median, range, recruiter, centre) and total length of appointment.[12] The qualitative component will thematically analyse the interviews using constant comparative techniques from grounded theory and will use a framework designed to incorporate concepts identified from the relevant literature.

The patient and recruiter interview transcriptions will be imported into the NVivo software package. It will then be analysed using Framework Analysis to address what the barriers and enablers to recruit to an RCT are and whether the trial documentation and recruitment process can be optimised to aid recruitment. We will explore additional themes as they emerge. A codebook will be developed to enable team-based analysis. The researchers will engage in a continuous process of reflexivity by documenting their own assumptions, viewpoints and impacts.

Ethics and dissemination

This study is sponsored by the UCL Joint Research Office (Reference number 125176). Ethical approval has been granted by the Greater Manchester West Research Ethics Committee (reference 20/NW/0071). The Health Research Authority have granted permission for the research to be conducted at the two NHS sites.

For peer review only

The sub-study has been prospectively registered with the Study Within A Trial (SWAT) database (reference SWAT 152).[12]

All investigators and trial site staff will comply with the requirements of General Data Protection Regulation with regard to the collection, storage, processing and disclosure of personal information and will uphold the Data Protection Act's core principles. A manuscript detailing the summative findings will be submitted to peer-reviewed journals for publication.

Acknowledgements

The authors would like to thank the Surgical Intervention Group (Sig) at University College London for their helpful comments on the drafts of this protocol.

Contributors: DW, SRM and JF designed the study. EB and DW drafted this manuscript. EB, VH, MS and ENL will conduct screening and data collection. Analysis will be performed by DW. All authors reviewed this manuscript for intellectual content and approved the final version. Mr Noam Tamir was the patient representative for the study and the authors would like to thank him for his insightful feedback and contribution.

Competing interests: The authors declare no competing interests.

Funding: This work was supported by the National Institute for Health research Award ID PB-PG-0817-20023.

Provenance and peer review: Not commissioned; peer reviewed for ethical and funding approval prior to submission.

Data sharing statement: Data sharing is not applicable as no datasets have been generated and/or analysed for this study. Results from this protocol will be published in a peer reviewed journal once complete. The data can also be requested by contacting the authors.

For peer review only

References

1 Canadian Task Force on the Periodic Health Examination. The periodic health examination. *Canadian Medical Association journal* 1979;**121**:1193–254.

2 Ross S, Grant A, Counsell C, *et al.* Barriers to participation in randomised controlled trials: a systematic review. *Journal of clinical epidemiology* 1999;**52**:1143–56. doi:10.1016/s0895-4356(99)00141-9

3 Prescott R, Counsell C, Gillespie W, *et al.* Factors that limit the quality, number and progress of randomised controlled trials. *Health technology assessment* 1993;**3**:1–143.

4 Hunninghake DB, Darby CA, Probstfield JL. Recruitment experience in clinical trials: Literature summary and annotated bibliography. *Controlled Clinical Trials* 1987;**8**:6–30. doi:10.1016/0197-2456(87)90004-3

5 Haidich A-B, Ioannidis JPA. Patterns of patient enrollment in randomized controlled trials. *Journal of Clinical Epidemiology* 2001;**54**:877–83. doi:10.1016/S0895-4356(01)00353-5

6 Donovan JL, Lane JA, Peters TJ, *et al.* Development of a complex intervention improved randomization and informed consent in a randomized controlled trial. *Journal of clinical epidemiology* 2009;**62**:29–36. doi:10.1016/j.jclinepi.2008.02.010

7 Mapstone J, Elbourne D, Roberts IG. Strategies to improve recruitment to research studies. In: Mapstone J, ed. *Cochrane Database of Systematic Reviews*. Chichester, UK: : John Wiley & Sons, Ltd 2007. doi:10.1002/14651858.MR000013.pub3

8 Donovan J. Quality improvement report: Improving design and conduct of randomised trials by embedding them in qualitative research: ProtecT (prostate testing for cancer and treatment) study * Commentary: presenting unbiased information to patients can be difficult. *BMJ* 2002;**325**:766–70. doi:10.1136/bmj.325.7367.766

9 Martin D, Koti R, Gurusamy K, *et al.* The cardiac output optimisation following liver transplant (COLT) trial: a feasibility randomised controlled trial. *HPB* 2020;**22**:1112–20. doi:10.1016/j.hpb.2019.11.011

10 Jenkins V, Fallowfield L. Reasons for accepting or declining to participate in randomized clinical trials for cancer therapy. *British Journal of Cancer* 2000;**82**:1783–8. doi:10.1054/bjoc.2000.1142

11 Paramasivan S, Strong S, Wilson C, *et al.* A simple technique to identify key recruitment issues in randomised controlled trials: Q-QAT - quanti-qualitative appointment timing. *Trials* 2015;**16**:88. doi:10.1186/s13063-015-0617-1

12 Wagstaff D, Moonesinghe R. SWAT 152: A qualitative investigation of patient recruitment to a perioperative feasibility randomised trial. 2020;:1–2.https://www.qub.ac.uk/sites/TheNorthernIrelandNetworkforTrialsMethodologyResearch/FileStore/Filetoupload,1203901,en.pdf (accessed 24 Nov 2021).

For peer review only

Appendices

Appendix 1: Study flowchart from RCT protocol (submitted for publication; manuscript ID: bmjopen-2021-055864.R1).

Provisional consent will be obtained while patient is on the waiting list. At admission confirmation of consent will be followed by enrolment, randomisation and treatment.

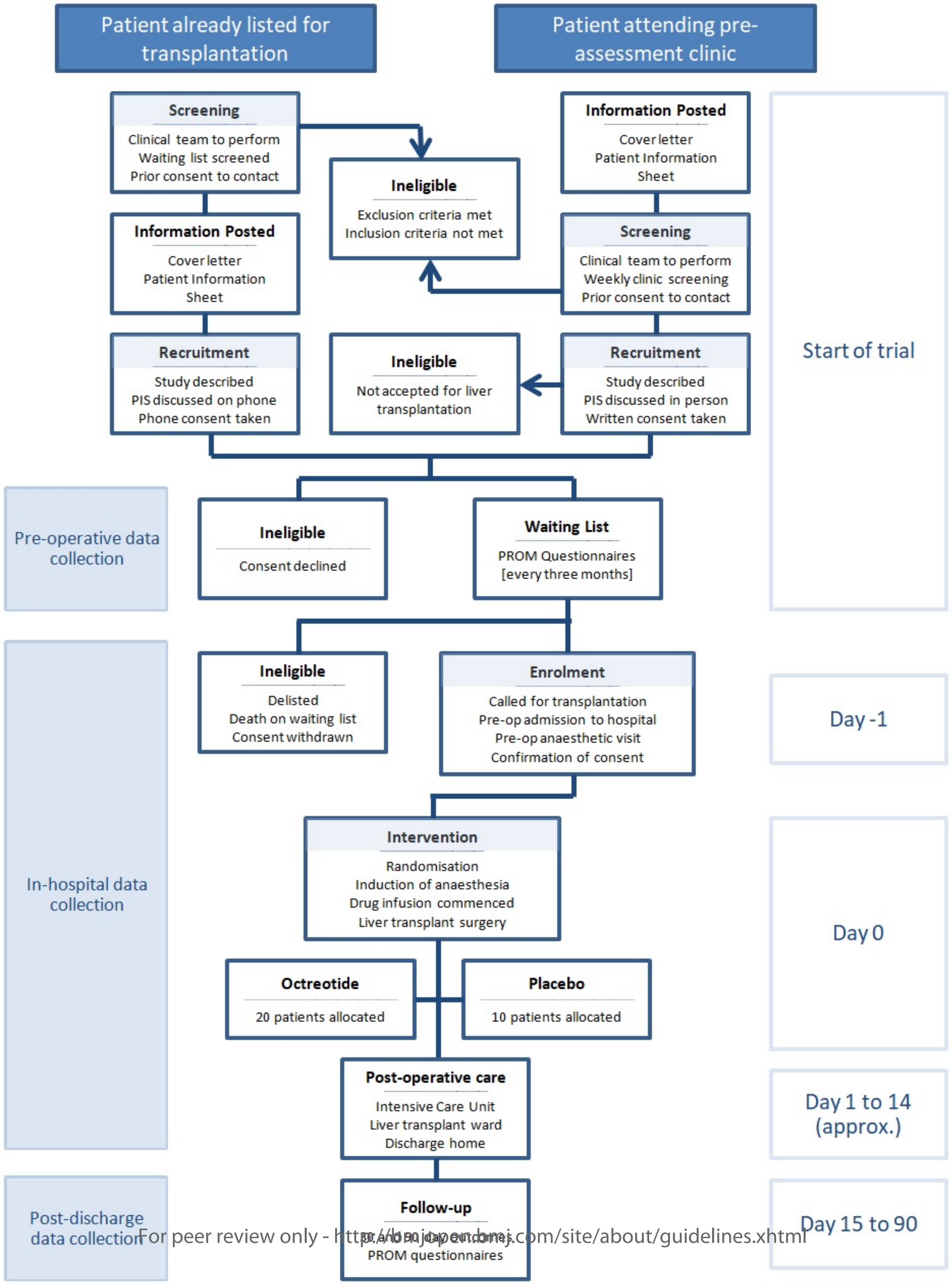

Appendix 2: Participant Information Sheet – for patients

Understanding patient recruitment to a perioperative RCT. A qualitative study.

Participant Information Sheet (Patients)

Introduction

We would like to invite you to participate in this sub-study exploring recruitment to a randomised controlled trial, 'Assessing the Impact of Octreotide Infusion during Liver Transplantation' (henceforth referred to as 'the Trial'). We are interested in learning about why patients are willing (or not) to participate in the Trial. To do this, we will invite all patients who are approached to participate in the Trial to complete a questionnaire. The questionnaire will explore views on the Trial and reasons for agreeing or declining to participate. If recruitment rates to the Trial drop below predetermined levels, or if the Trial team have concerns about the recruitment process, we will seek permission from patients to record recruitment consultations and interview them afterwards by telephone. Recorded interviews will be transcribed (written up) and the tape will then be wiped clean. We will also interview members of the Trial team. Any contributions you make will be anonymised and collated as part of a larger group.

Why is this study being done?

We hope to understand and improve processes for recruiting patients to the Trial.

What will happen if I complete a Questionnaire?

After discussing the Trial with the study team (your 'recruitment consultation'), you will be invited to complete and return a questionnaire. The questionnaire will explore your views on the Trial and reasons for agreeing or declining to participate. All information gathered will be treated as confidential by the study personnel and anonymised for analysis.

What will happen if I take part in the recording of recruitment consultations and subsequent Interviews?

If you are asked, and consent for us to do so, we will use a digital voice recorder during your recruitment consultation. We will use this recording to analyse how the Trial was discussed with you. At least 24 hours later, we will conduct a telephone interview with you to ask you about your views of the Trial, the recruitment process, and your reasons for accepting or declining to participate. The interview will be recorded. All information gathered will be treated as confidential by the study personnel and anonymised for analysis. We will use our findings to help the Trial team improve their recruitment processes.

How long will the study last?

The total duration of the study will be two years but your involvement will only be for the questionnaire you submit, or for the consultation recording and subsequent interview.

Can I stop being in the study?

For peer review only

You can decide to stop participating at any time. After a questionnaire, recruitment consultation or interview has been concluded, you are free to decline consent to any future involvement in the study but historic data will not be destroyed.

What risks can I expect from being in the study?

This is a very low risk study. Information you provide about your experiences and opinions will be documented, but your name will not be used in any reports of the information provided. The information obtained from these observations will only be used by the project researchers and will be locked at our project offices. Hard copies of research data will be shredded after 10 years, and securely disposed of in confidential waste. We will do our best to make sure that any personal information gathered for this research study is kept private and treated in accordance with the Data Protection Act 2018 (https://www.gov.uk/government/collections/data-protection-act-2018).

Are there benefits to taking part in the study?

There will be no direct benefit to you from participating in this study. However, the information that you provide will help the Trial team and future researchers understand and improve recruitment to surgical and perioperative trials which we hope will benefit future groups of patients.

What other choices do I have if I do not take part in this study?

You are free to choose not to participate in the study. If you decide not to take part in this study, there will be no penalty to you, it will not affect your clinical care or your participation in the Trial.

What are the costs of taking part in this study? Will I be paid for taking part in this study?

There are no costs to you for taking part in this study. You will not be paid for taking part in this study.

What are my rights if I take part in this research study?

Taking part in this study is your choice. You may choose either to take part or not to take part in the study. No matter what decision you take, there will be no penalty to you in any way.

Who can answer my questions about the research study?

You can talk to the researchers about any questions or concerns you have about this study. Their contact details can be found below. If for any reason you do not wish to do this, please see the section below.

Giving consent to participate in the research study

You may keep this information sheet if you wish. Participation in this study is voluntary. You have the right to decline to participate in the study without penalty. If you do not wish to participate, you should inform the researcher when given a questionnaire, or at the beginning of the recruitment consultation/interview. If you do

For peer review only

not agree to quotes or other results arising from your participation in the study being included, even anonymously, in reports about the study, please tell the researcher.

Who is organising and funding the research study?

The study is organised by the Royal College of Anaesthetists and led by Prof SR Moonesinghe, a Consultant in Intensive Care Medicine and Anaesthesia at UCLH, and Professor of Anaesthesia and Perioperative Medicine at UCL. Prof Moonesinghe is assisted by a multidisciplinary project team consisting of anaesthetists, surgeons and patients. The research costs for the study have been supported by a grant from the National Institute for Health Research.

Who has reviewed the research study?

The study design has been reviewed by the UCL Research Ethics Committee and the Health Research Authority before any patients or staff were approached to participate.

What will happen to the results?

The results will be analysed and used to help improve processes for recruiting patients to the Trial. In addition, results will be written up for publication in scientific journals and for presentation at conferences. All information gathered will be anonymised and will not be traceable to you.

"What if there is a problem" or "What happens if something goes wrong?"

If you wish to complain, or have any concerns about any aspect of the way you have been approached or treated by members of staff you may have experienced due to your participation in the research, please contact the research team. UCL complaints mechanisms may also be available to you, or the Patient Advice and Liaison Service (PALS) at your hospital (see below).

Chief Investigator: Prof SR Moonesinghe **email:** ramani.moonesinghe@nhs.net

Researcher: Dr Duncan Wagstaff **email:Duncan.wagstaff@nhs.net**

UCL Centre for Perioperative Medicine, Charles Bell House, 43-45 Foley Street, London, W1W 7TS

For peer review only

Appendix 3: Participant Information Sheet – for recruiters

Understanding patient recruitment to a perioperative RCT. A qualitative study.

Participant Information Sheet (Recruiters)

Introduction

We would like to invite you to participate in this sub-study exploring recruitment to a randomised controlled trial, 'Assessing the Impact of Octreotide Infusion during Liver Transplantation' (henceforth referred to as 'the Trial'). We are interested in learning about why patients are willing (or not) to participate in the Trial. To do this, we are surveying and interviewing patients, recording recruitment consultations, and also interviewing members of the Trial team. Any contributions you make will be anonymised and collated as part of a larger group.

Why is this sub-study being done?

We hope to understand and improve processes for recruiting patients to the Trial.

What will happen if I take part in the interviews?

You may be sent an email and subsequently approached by a researcher to invite you take part in an interview. This participant information sheet will be provided in advance of you being interviewed, and with sufficient time (at least 24 hours) to be able to ask any questions you may have. Having had your questions answered satisfactorily, you will be asked to sign a consent form at the start of the interview. The interview will be conducted in private and last approximately 30-60minutes during which time we will ask you some questions about the Trial, its recruitment processes and how you think they might be improved. We will take notes of the discussion and an audio recording will also be made using a digital voice recorder. Recorded interviews will be transcribed (written up) and the tape will then be wiped clean. All information gathered will be treated as confidential by the study personnel and anonymised for analysis.

How long will the sub-study last?

The total duration of the study will be two years but your involvement will only be for your interview.

Can I stop being in the sub-study?

You can decide to stop participating at any time. After an interview has been concluded, you are free to decline consent to any future involvement in the study but historic data will not be destroyed.

What risks can I expect from being in the sub-study?

This is a very low risk study. Information you provide about your experiences and opinions will be documented, but your name will not be recorded or used in any reports of the information provided. The information obtained from these interviews will only be used by the project researchers and will be locked at our project offices. Hard copies of research data will be shredded after 10 years, and securely disposed of in confidential

For peer review only

waste. We will do our best to make sure that any personal information gathered for this research study is kept private and treated in accordance with the Data Protection Act 2018 (https://www.gov.uk/government/collections/data-protection-act-2018).

Are there benefits to taking part in the sub-study?

There will be no direct benefit to you from participating in this study. However, the information that you provide will help the Trial team and future researchers understand and improve recruitment to surgical and perioperative trials.

What other choices do I have if I do not take part in this sub-study?

You are free to choose not to participate in the study. If you decide not to take part in this study, there will be no penalty to you.

What are the costs of taking part in this study? Will I be paid for taking part in this sub-study?

There are no costs to you for taking part in this study. You will not be paid for taking part in this study.

What are my rights if I take part in this research sub-study?

Taking part in this study is your choice. You may choose either to take part or not to take part in the study. No matter what decision you take, there will be no penalty to you in any way.

Who can answer my questions about the sub-study?

You can talk to the researchers about any questions or concerns you have about this study. Their contact details can be found below. If for any reason you do not wish to do this, please see the section below.

Giving consent to participate in the sub-study

You may keep this information sheet if you wish. Participation in this study is voluntary. You have the right to decline to participate in the study without penalty. If you do not wish to participate, you should inform the researcher when given a questionnaire, or at the beginning of the recruitment consultation/interview. If you do not agree to quotes or other results arising from your participation in the study being included, even anonymously, in reports about the study, please tell the researcher.

Who is organising and funding the sub-study?

For peer review only

The study is by Prof SR Moonesinghe, a Consultant in Intensive Care Medicine and Anaesthesia at UCLH, and Professor of Anaesthesia and Perioperative Medicine at UCL. Prof Moonesinghe is assisted by a multidisciplinary project team consisting of anaesthetists, surgeons and patients. The research costs for the study have been supported by a grant from the National Institute for Health Research.

Who has reviewed the sub-study?

The sub-study has been reviewed by the UCL Research Ethics Committee and the Health Research Authority before any patients or staff were approached to participate.

What will happen to the results?

The results will be analysed and written up for publication in scientific journals and for presentation at conferences. All information gathered will be anonymised and will not be traceable to you.

"What if there is a problem" or "What happens if something goes wrong?"

If you wish to complain, or have any concerns about any aspect of the way you have been approached or treated by members of staff you may have experienced due to your participation in the research, please contact the research team. UCL complaints mechanisms may also be available to you.

Chief Investigator: Prof SR Moonesinghe **email:** ramani.moonesinghe@nhs.net

Researcher: Dr Duncan Wagstaff **email:Duncan.wagstaff@nhs.net**

[illegible]

For peer review only

Appendix 4: Consent Form

CONSENT FORM   Participant Identification Number:

Title of Project: **Understanding Patient Recruitment to a Perioperative RCT**

Chief Investigator: **Dr Ramani Moonesinghe**

Please initial box

I confirm that I have read and understand the Participant Information Sheet (version 1.2) for the above study. I have had the opportunity to consider the information, ask questions and have had these answered satisfactorily ☐

I understand that my participation in an interview or recording of recruitment consultation is voluntary and that I am free to withdraw at any time without giving any reason, without my employment or legal rights being affected. ☐

I understand that data and quotations I provide may be used (anonymised fully) in future publications of this research. ☐

I understand that audio recordings of the interviews and/or recruitment consultations will be made and stored anonymously. The recording will be deleted once it has been written up. ☐

☐

For peer review only

I understand that, in the event of my withdrawal from the study, data I provide prior to my withdrawal will be retained (anonymised fully) for analysis and publication.

I understand that data collected during the study may be looked at by individuals from University College London (UCL) or from regulatory authorities where it is relevant to my taking part in this research. I give permission for these individuals to have access to my records. Information will be treated as strictly confidential and handled in accordance with the provisions of the General Data Protection Regulation. ☐

I agree to take part in the above study. ☐

Name of Participant	Date	Signature
Name of Person taking consent	Date	Signature

When completed: 1 for participant; 1 (original) for researcher site file

For peer review only

Appendix 5: Patient Questionnaire

Clinical Trials Questionnaire

We are interested in the reasons why patients accept or decline to take park in the clinical trial of octreotide infusion during liver transplantation. We would be grateful if you could complete and this questionnaire. It will not be shown to your doctor or any of the staff at the hospital.

	Yes	No	Not decided yet
Did you agree to take part in the trial mentioned above?			

Below are some reasons that may have influenced your decision to accept or decline to take part in this trial. Please answer each question by ticking the box that shows most clearly how you feel.

	Strongly agree	Agree to some extent	Unsure	Disagree to some extent	Strongly disagree
1) I thought the trial/study offered the best treatment available.					
2) I believed the benefits of treatment in the trial/study would out-weigh any side-effects.					
3) I was satisfied that either treatment in the trial/study would be suitable for me.					
4) I was worried that my illness would get worse unless I joined the trial/study.					
5) The idea of randomisation worried me.					

For peer review only

6) I wanted the doctor to choose my treatment rather than be randomised by computer					
7) The doctor told me what I needed to know about the trial					
8) I trusted the doctor treating me.					
9) I was given too much information to read about the trial.					
10) I was given enough information to read about the trial.					
11) I knew that I could leave the trial at any time and still be treated.					
12) I did not feel able to say no.					
13) I wanted to help with the doctors' research.					
14) I feel that others will benefit from the results of the trial.					
15) The doctor wanted me to join the trial.					
16) Others (e.g. family/friends) wanted me to join the trial.					

Which as the most important reason for you out of this list? (Please give number)________________

Are there any other reasons for your decision? Please list them below

Thank you very much for completing this questionnaire.

For peer review only

Appendix 6: Interview Topic Guide – for patients

Understanding Patient Recruitment to a Perioperative RCT.

INTERVIEW TOPIC GUIDE

1. Did you agree to take part in the study?
2. What were your reasons for agreeing/declining to take part?
3. How did you feel about the written information you received before speaking to the study team?
4. After speaking to the study team, did you understand everything that you wanted to about the trial?
5. What did you understand the potential benefits of participation might be?
6. What did you understand the potential harms of participation might be?
7. Was there any other information that you wanted to know before making your decision?
8. How did you feel about the process of randomisation?
9. Did discussions with family/friends affect your reasons for agreeing/declining to take part in the trial, and if so, in which ways?
10. Did your doctor(s) want you to take part in the trial?
11. Is there anything else that you'd like to mention?

For peer review only

Appendix 7: Interview Topic Guide – for recruiters

Understanding Patient Recruitment to a Perioperative RCT.

INTERVIEW TOPIC GUIDE - recruiters

1. Please describe your role in the study

2. How do you feel about the **written information** about the trial which is provided for participants before they are recruited? (i.e. timing, format, content)

3. How do you feel about the processes for recruiting patients?
   a. Location
   b. Timings
   c. Information sheets
   d. Consent form

4. What do you think potential participants understand the potential **benefits** of participation might be?

5. What do you think potential participants understand the potential **risks** of participation might be?

6. What are the main reasons you think that patients **agree** to take part?

7. What are the main reasons you think that patients **decline** to take part?

8. Are there any aspects of the trial which are particularly hard to explain to participants?

9. How do you think patients feel about the process of randomisation?

10. Do you think discussions with family/friends affect patients' decisions for agreeing/declining to take part in the trial, and if so, in which ways?

11. How do you think the recruitment process(es) could be improved?

12. Is there anything else that you'd like to mention?

For peer review only

Understanding recruitment to a perioperative randomised controlled trial: protocol for a mixed-methods sub-study nested within a feasibility trial of octreotide infusion during liver transplantation

Journal:	*BMJ Open*
Manuscript ID	bmjopen-2021-060177.R1
Article Type:	Protocol
Date Submitted by the Author:	03-May-2022
Complete List of Authors:	Brodkin, Edgar; Royal Free London NHS Foundation Trust, Department of Anaesthesia<br>Loh, Ee-Neng; Royal Free London NHS Foundation Trust, Department of Anaesthesia<br>Spiro, Michael; Royal Free London NHS Foundation Trust, Department of Anaesthesia; University College London, Department of Surgical Biotechnology, Division of Surgery and Interventional Science<br>Hannon, Vivienne; Royal Free London NHS Foundation Trust, Department of Anaesthesia<br>Fabes, Jez; Peninsula Medical School; Royal Free London NHS Foundation Trust, Department of Anaesthesia<br>Moonesinghe, S. Ramani; University College London, Centre for Perioperative Medicine, Research Department of Targeted Intervention<br>Wagstaff, Duncan; University College London, Centre for Perioperative Medicine, Research Department of Targeted Intervention
**Primary Subject Heading**:	Qualitative research
Secondary Subject Heading:	Research methods
Keywords:	QUALITATIVE RESEARCH, STATISTICS & RESEARCH METHODS, Transplant surgery < SURGERY, Clinical trials < THERAPEUTICS, TRANSPLANT SURGERY

Understanding recruitment to a perioperative randomised controlled trial: protocol for a mixed-methods sub-study nested within a feasibility trial of octreotide infusion during liver transplantation

E Brodkin[1], EN Loh[1], M Spiro[1,2], V Hannon[1], J Fabes[1,3], SR Moonesinghe[4], D Wagstaff[4]

[1] Department of Anaesthesia, Royal Free London NHS Foundation Trust, London, United Kingdom
[2]Department of Surgical Biotechnology, Division of Surgery and Interventional Science, University College London, United Kingdom
[3] Peninsula Medical School, University of Plymouth, Plymouth, United Kingdom
[4]Centre for Perioperative Medicine, Research Department of Targeted Intervention, Division of Surgery and Interventional Science, University College London, London, United Kingdom

Email addresses of authors:

E Brodkin – edgarbrodkin@nhs.net
EN Loh - ee-neng.loh@nhs.net
M Spiro - michaelspiro@nhs.net
V Hannon - vivienne.hannon@nhs.net
J Fabes - jeremy.fabes@nhs.net
SR Moonesinghe - ramani.moonesinghe@nhs.net
D Wagstaff - duncan_wagstaff@yahoo.co.uk

Correspondence to: Dr Duncan Wagstaff, email duncan_wagstaff@yahoo.co.uk, mobile number +447812125650.
Centre for Perioperative Medicine, Department for Targeted Intervention, Division of Surgery and Interventional Science, University College London, Charles Bell House, 43-47 Foley St, London W1T 7AA UK.

Word count (excluding title page, abstract, references, footnotes, figures and tables): 1955

For peer review only

Abstract

Introduction Recruitment to perioperative randomised controlled trials is known to be challenging. Qualitative methods offer insight into barriers and enablers to participation. This is a sub-study within a feasibility randomised controlled trial of octreotide infusion during liver transplantation at two NHS hospitals, which will evaluate patient and staff experiences of trial processes. By sharing formative understanding from these methods with the trials team we aim to improve staff-patient interactions and hence recruitment rates.

Methods and Analysis This prospective mixed-methods study will comprise two workstreams. First, after consent to the randomised controlled trial is sought, all patients will be invited to complete a questionnaire to explore their perceptions of the information given to them and motivating factors that influenced their decision to consent or not. Questionnaires will be analysed using descriptive statistics and Framework Analysis.

If the recruitment:approach ratio drops below a predetermined ratio or if there are any specific recruitment concerns from the trials team, a second workstream involving mixed-methods fieldwork will be implemented. This will involve audio-recording of recruitment consultations and a follow-up semi-structured interview to explore patients' perception of their decision-making regarding recruitment. Semi-structured interviews will also be conducted with the recruitment team to establish their views about the trial, barriers to recruitment and ways to overcome them. Recruitment consultations will be analysed using Q-QAT methodology and interviews will be analysed using Framework Analysis. Findings from both workstreams will be formatively fed back to the trials team to enable iterative improvement to recruitment processes.

Ethics and Dissemination Approval has been granted by Greater Manchester West Research Ethics Committee (ref 20/NW/0071), the Health Research Authority and the local Research and Development offices. A manuscript detailing the summative findings will be submitted to peer-reviewed journals.

Registration The study has been registered with the Study Within a Trial database (ref 152).

Key Words Qualitative research, statistics and research methods, transplant surgery, clinical trials.

For peer review only

Article Summary

Strengths and limitations of this study

- The use of qualitative methodologies will give us in-depth insight into the barriers and enablers to recruitment to this perioperative randomised controlled trial (RCT)
- Data gathered across two sites will make the findings more generalisable to other centres
- Formative feedback to the RCT team during ongoing recruitment will allow modification of the recruitment process to improve recruitment rates
- The role of the two of the researchers (EB, DW) as anaesthetists and members of the RCT trial management group (TMG) may affect the conduct of the recruitment team interviews, although they will not be performing the interviews themselves

For peer review only

Introduction

Multicentre, double-blind RCTs are the methodology of choice for investigating the effectiveness and safety of healthcare interventions.[1] Previous research has shown that recruitment to RCTs can be challenging.[2] Barriers to participation may include, for example, concern over the concept of randomisation, incomplete explanations of trial methodology, or a lack of balance in the way that treatment arms are explained to potential participants by research staff.[2] These difficulties in recruitment can result in failing to start, abandoning or revising target sample sizes of RCTs.[3–5] Furthermore, in multicentre trials, poor or unequal recruitment can negatively impact on staff morale, equality of workload and trial costs in addition to introducing bias and reducing statistical power.[6]

Previous systematic reviews have shown that methods implemented to improve recruitment to research studies in general have not shown clear benefit. Multicentre trials are also under-represented in this literature.[7] However, Donovan et al.[6,8] have demonstrated how contemporaneous qualitative research methods can improve the rates of randomisation and informed consent in multicentre RCTs, including in the perioperative setting. They described how with improved understanding of recruitment processes (both 'as planned', and 'as done'), as well as patient perceptions of recruitment, can be formatively fed back to trial teams to enable timely adjustments to be made. Rooshenas et al.[9] have further demonstrated how these interventions can support recruitment in several UK multicentre RCTs. These included one RCT with a placebo arm, such as in our RCT, which can present additional challenges to recruitment such as difficulty for recruiters in articulating the placebo arm.

This mixed-methods sub-study is nested within a double-blind randomised, placebo-controlled feasibility trial investigating the use of octreotide infusion during liver transplantation at two NHS hospitals. Octreotide infusions are currently used during liver transplantation in some centres to potentially reduce bleeding, improve renal outcomes and improve haemodynamic status. However, this practice is based on observational studies. This trial (henceforth referred to as 'the RCT') will be the first to assess the feasibility of randomising patients undergoing liver transplantation to receive either octreotide infusion or placebo. The protocol for the RCT has already been submitted for publication (Manuscript ID: bmjopen-2021-055864.R1).

Consent for participation and recruitment into the RCT will be sought when patients come for an inpatient assessment prior to being placed on the transplant waiting list. Patients already on the waiting list will be contacted by telephone. As there can be a prolonged time period on the transplant waiting list, consent will be confirmed ('enrolment') on admission to hospital for their transplant (Appendix 1). This strategy has been successfully implemented in comparable interventional studies in this population.[10] The focus of our study will be the initial recruitment stage.

For peer review only

Aims & Objectives

Aim

To evaluate the barriers and enablers to recruitment to the RCT.

Objectives

1. To survey patients' reasons and motivation for participation in the RCT using a written questionnaire
2. In the event of low recruitment rates, to undertake in-depth mixed-methods evaluation of consultation recordings and patient/staff interviews with regards to the recruitment processes
3. To provide formative and summative feedback to the RCT team, which will enable necessary adjustments to the recruitment process

Research Questions

1. What are the barriers and enablers to patient recruitment to this RCT?
2. Can the recruitment processes be optimised to improve recruitment throughout the feasibility study or for any follow-on substantive trials?

For peer review only

Methods and Analysis

Study Design

This is a nested mixed-methods sub-study of a feasibility RCT exploring the use of an octreotide infusion during orthotopic liver transplantation across two centres; the Royal Free Hospital (RFH), and University Hospital Birmingham (UHB). The RFH will be the lead site where the team who led the grant application and study design are based.

This sub-study comprises two workstreams: a questionnaire of all patients approached for recruitment to the RCT; and mixed-methods fieldwork. The mixed-methods fieldwork will only be undertaken if the observed recruitment:approach ratio is below predetermined thresholds of <0.3 at the RFH, or < 0.15 at University Hospitals Birmingham (UHB), after at least 12 patients at each site, or if the RCT team have specific concerns about recruitment processes. The predetermined thresholds have been chosen to reflect recruitment rates during a previous RCT in this patient population at the RFH,[10] and lower recruitment rates at secondary centres.

Interim findings from the sub-study will be fed back to the Trial Management Group (TMG) of the RCT at fortnightly meetings. This will enable us to pick up and respond to themes we identify both during the feasibility study and potentially in our planned full randomised trial and will enable the TMG to make timely adjustments to recruitment processes where needed. These will depend on the issues identified, but may include written guidance, confidential feedback or additional training. Summative findings will be provided to the various stakeholders including the patient representative and the clinical teams.

Patient and public involvement

The patient representative of the TMG, who has experience with both liver transplantation and recruitment to research studies, was involved in reviewing and refining study design and methodology. Their feedback was used to draft and amend study documentation. They will continue to be involved for the duration of the study and will have input into the dissemination of both the formative and summative findings.

Eligibility and consent

All patients eligible for the RCT will be eligible for recruitment to the sub-study. Completion of the questionnaire will be taken as implied consent. Participants who are approached for recruitment to the RCT, during their inpatient assessment or via telephone, will be provided with a written patient information sheet (PIS) together with the contact details of the recruitment team (Appendix 2). They will be given at least 24 hours to review the PIS prior to

For peer review only - http://bmjopen.bmj.com/site/about/guidelines.xhtml

their recruitment consultation. The sub-study will be discussed with them (either in person during the admission for inpatient assessment or by telephone) prior to the recruitment consultation.

If the mixed-methods fieldwork is initiated, all members of the recruitment team and all patients approached for trial participation will be eligible for recruitment. Individuals will only be excluded if they refuse to provide written consent or if they do not speak English. The recruitment team will also be given at least 24 hours to review the PIS prior to their interviews (Appendix 3).

Recruitment

All patients approached for consent to the feasibility RCT will be invited to complete the questionnaire after that consultation. The RCT aims to *enrol* 30 patients within 10 months but will likely have to *recruit* a far greater number of patients to achieve this as not all patients on the liver transplant waiting list will receive a graft organ within the recruitment timeframe (Appendix 1). Recruitment is due to start from May 2022. There is therefore no specific targeted sample size for the questionnaires; this phase will conclude when recruitment to the RCT ends.

If the mixed-methods fieldwork workstream is triggered, subsequent consecutive patients will be asked for consent (Appendix 4) to have their recruitment consultation (face to face or telephone) recorded and then be interviewed afterwards. Recruitment will continue until theoretical saturation has been achieved; this is likely to occur after approximately 12 patients have been recruited. Recruiters at both sites will be interviewed until theoretical saturation has been achieved (likely to be 6 recruiters).

Data Collection

The questionnaire is based on a previously validated questionnaire used in a similar scenario.[11] It explores patients' perceptions of the trial information given to them and their rationale behind agreeing or declining to consent for the RCT. This has been adapted for our study with input from a patient expert on the TMG (Appendix 5). For the patients contacted via telephone, questionnaires will be completed in hard copy and returned to the study team.

The fieldwork will comprise three different approaches: audio recordings of the recruitment consultation, patient interviews and recruiter interviews.

Audio recordings of face-to-face and telephone recruitment consultations to the RCT will be taken via an encrypted digital voice recorder. Semi-structured interviews with patients will be conducted by telephone at least 24 hours after the recruitment consultation. This will be

For peer review only

recorded using an encrypted digital voice recorder. The interviews will focus on the patients' perception of the information they were given about the recruitment consultation and the rationale behind their decision to consent to the RCT or not.

Semi-structured interviews with members of the research team will be conducted in person or via telephone and recorded on an encrypted digital voice recorder and professionally transcribed.

All interviews will be performed by experienced interviewers who are not members of the trial TMG and do not undertake trial recruitment. As EB and DW are members of the trial TMG they will not be performing the interviews. All recordings will be professionally transcribed. Interview Topic Guides (Appendices 6 and 7) will be used to ensure consistency and will be updated iteratively based on feedback from the TMG.

Data Analysis

Questionnaires will be analysed formatively every 20 patients approached and summatively at the end of the feasibility study. Responses to closed questions will be summarised using descriptive statistics and thematic analysis for any open-ended answers.

Audio recordings of recruitment consultations will be analysed according to the Q-QAT methodology:[12] this involves summarising recruitment consultations both quantitatively and qualitatively. The quantitative component records the time taken to present each of the RCT treatments (mean, median range; recruiter; centre), the time taken to explain the design, purpose and procedures of the RCT (mean, median, range, recruiter, centre) and total length of appointment.[13] The qualitative component will thematically analyse the interviews using constant comparative techniques from grounded theory and will use a framework designed to incorporate concepts identified from the relevant literature.

The patient and recruiter interview transcriptions will be imported into the NVivo software package. It will then be analysed using Framework Analysis to address what the barriers and enablers to recruit to an RCT are and whether the trial documentation and recruitment process can be optimised to aid recruitment. We will explore additional themes as they emerge. A codebook will be developed to enable team-based analysis. The researchers will engage in a continuous process of reflexivity by documenting their own assumptions, viewpoints and impacts.

As delineated in Rooshenas et al.[14], a key set of recruitment issues will be devised, triangulating the data from the aforementioned analyses and also quantitative data from the screening log. These will be presented to the Chief Investigator and at the fortnightly TMG meetings.

For peer review only

Ethics and dissemination

This study is sponsored by the UCL Joint Research Office (Reference number 125176). Ethical approval has been granted by the Greater Manchester West Research Ethics Committee (reference 20/NW/0071). The Health Research Authority have granted permission for the research to be conducted at the two NHS sites.

The sub-study has been prospectively registered with the Study Within A Trial (SWAT) database (reference SWAT 152).[12]

All investigators and trial site staff will comply with the requirements of General Data Protection Regulation with regard to the collection, storage, processing and disclosure of personal information and will uphold the Data Protection Act's core principles. A manuscript detailing the summative findings will be submitted to peer-reviewed journals for publication.

Acknowledgements

The authors would like to thank the Surgical Intervention Group (Sig) at University College London for their helpful comments on the drafts of this protocol. Mr Noam Tamir was the patient representative for the study and the authors would like to thank him for his insightful feedback and contribution.

Contributors: DW, SRM and JF designed the study. EB and DW drafted this manuscript. EB, VH, MS and ENL will conduct screening and data collection. Analysis will be performed by DW. All authors reviewed this manuscript for intellectual content and approved the final version.

Competing interests: The authors declare no competing interests.

Funding: This work was supported by the National Institute for Health research Award ID PB-PG-0817-20023.

Provenance and peer review: Not commissioned; peer reviewed for ethical and funding approval prior to submission.

Data sharing statement: Data sharing is not applicable as no datasets have been generated and/or analysed for this study. Results from this protocol will be published in a peer reviewed journal once complete. The data can also be requested by contacting the authors.

For peer review only

References

1 Canadian Task Force on the Periodic Health Examination. The periodic health examination. *Can Med Assoc J* 1979;**121**:1193–254.

2 Ross S, Grant A, Counsell C, *et al.* Barriers to participation in randomised controlled trials: a systematic review. *J Clin Epidemiol* 1999;**52**:1143–56. doi:10.1016/s0895-4356(99)00141-9

3 Prescott R, Counsell C, Gillespie W, *et al.* Factors that limit the quality, number and progress of randomised controlled trials. *Health Technol Assess (Rockv)* 1993;**3**:1–143.

4 Hunninghake DB, Darby CA, Probstfield JL. Recruitment experience in clinical trials: Literature summary and annotated bibliography. *Controlled Clinical Trials* 1987;**8**:6–30. doi:10.1016/0197-2456(87)90004-3

5 Haidich A-B, Ioannidis JPA. Patterns of patient enrollment in randomized controlled trials. *Journal of Clinical Epidemiology* 2001;**54**:877–83. doi:10.1016/S0895-4356(01)00353-5

6 Donovan JL, Lane JA, Peters TJ, *et al.* Development of a complex intervention improved randomization and informed consent in a randomized controlled trial. *J Clin Epidemiol* 2009;**62**:29–36. doi:10.1016/j.jclinepi.2008.02.010

7 Mapstone J, Elbourne D, Roberts IG. Strategies to improve recruitment to research studies. In: Mapstone J, ed. *Cochrane Database of Systematic Reviews*. Chichester, UK: : John Wiley & Sons, Ltd 2007. doi:10.1002/14651858.MR000013.pub3

8 Donovan J. Quality improvement report: Improving design and conduct of randomised trials by embedding them in qualitative research: ProtecT (prostate testing for cancer and treatment) study * Commentary: presenting unbiased information to patients can be difficult. *BMJ* 2002;**325**:766–70. doi:10.1136/bmj.325.7367.766

9 Rooshenas L, Scott LJ, Blazeby JM, *et al.* The QuinteT Recruitment Intervention supported five randomized trials to recruit to target: a mixed-methods evaluation. *Journal of Clinical Epidemiology* 2019;**106**:108–20. doi:10.1016/j.jclinepi.2018.10.004

10 Martin D, Koti R, Gurusamy K, *et al.* The cardiac output optimisation following liver transplant (COLT) trial: a feasibility randomised controlled trial. *HPB* 2020;**22**:1112–20. doi:10.1016/j.hpb.2019.11.011

11 Jenkins V, Fallowfield L. Reasons for accepting or declining to participate in randomized clinical trials for cancer therapy. *British Journal of Cancer* 2000;**82**:1783–8. doi:10.1054/bjoc.2000.1142

For peer review only

12 Paramasivan S, Strong S, Wilson C, *et al.* A simple technique to identify key recruitment issues in randomised controlled trials: Q-QAT - quanti-qualitative appointment timing. *Trials* 2015;**16**:88. doi:10.1186/s13063-015-0617-1

13 Wagstaff D, Moonesinghe R. SWAT 152: A qualitative investigation of patient recruitment to a perioperative feasibility randomised trial. 2020;:1–2.https://www.qub.ac.uk/sites/TheNorthernIrelandNetworkforTrialsMethodologyResearch/FileStore/Filetoupload,1203901,en.pdf (accessed 24 Nov 2021).

14 Rooshenas L, Paramasivan S, Jepson M, *et al.* Intensive Triangulation of Qualitative Research and Quantitative Data to Improve Recruitment to Randomized Trials: The QuinteT Approach. *Qualitative Health Research* 2019;**29**:672–9. doi:10.1177/1049732319828693

For peer review only

Appendices

Appendix 1: Study flowchart from RCT protocol (submitted for publication; manuscript ID: bmjopen-2021-055864.R1).

Provisional consent will be obtained while patient is on the waiting list. At admission confirmation of consent will be followed by enrolment, randomisation and treatment.

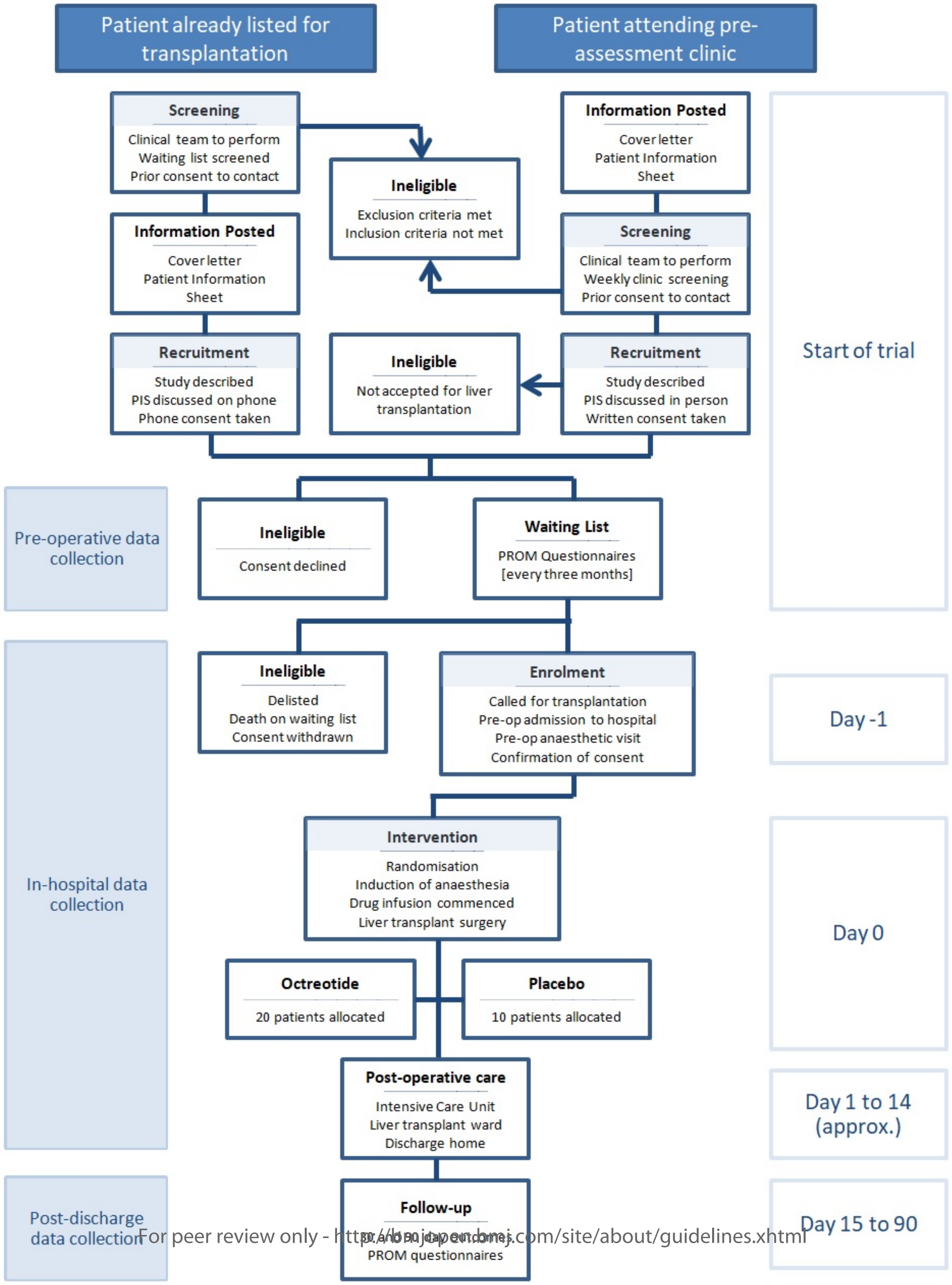

For peer review only - http://bmjopen.bmj.com/site/about/guidelines.xhtml

Appendix 2: Participant Information Sheet – for patients

Understanding patient recruitment to a perioperative RCT. A qualitative study.

Participant Information Sheet (Patients)

Introduction

We would like to invite you to participate in this sub-study exploring recruitment to a randomised controlled trial, 'Assessing the Impact of Octreotide Infusion during Liver Transplantation' (henceforth referred to as 'the Trial'). We are interested in learning about why patients are willing (or not) to participate in the Trial. To do this, we will invite all patients who are approached to participate in the Trial to complete a questionnaire. The questionnaire will explore views on the Trial and reasons for agreeing or declining to participate. If recruitment rates to the Trial drop below predetermined levels, or if the Trial team have concerns about the recruitment process, we will seek permission from patients to record recruitment consultations and interview them afterwards by telephone. Recorded interviews will be transcribed (written up) and the tape will then be wiped clean. We will also interview members of the Trial team. Any contributions you make will be anonymised and collated as part of a larger group.

Why is this study being done?

We hope to understand and improve processes for recruiting patients to the Trial.

What will happen if I complete a Questionnaire?

After discussing the Trial with the study team (your 'recruitment consultation'), you will be invited to complete and return a questionnaire. The questionnaire will explore your views on the Trial and reasons for agreeing or declining to participate. All information gathered will be treated as confidential by the study personnel and anonymised for analysis.

What will happen if I take part in the recording of recruitment consultations and subsequent Interviews?

If you are asked, and consent for us to do so, we will use a digital voice recorder during your recruitment consultation. We will use this recording to analyse how the Trial was discussed with you. At least 24 hours later, we will conduct a telephone interview with you to ask you about your views of the Trial, the recruitment process, and your reasons for accepting or declining to participate. The interview will be recorded. All information gathered will be treated as confidential by the study personnel and anonymised for analysis. We will use our findings to help the Trial team improve their recruitment processes.

How long will the study last?

The total duration of the study will be two years but your involvement will only be for the questionnaire you submit, or for the consultation recording and subsequent interview.

Can I stop being in the study?

For peer review only

You can decide to stop participating at any time. After a questionnaire, recruitment consultation or interview has been concluded, you are free to decline consent to any future involvement in the study but historic data will not be destroyed.

What risks can I expect from being in the study?

This is a very low risk study. Information you provide about your experiences and opinions will be documented, but your name will not be used in any reports of the information provided. The information obtained from these observations will only be used by the project researchers and will be locked at our project offices. Hard copies of research data will be shredded after 10 years, and securely disposed of in confidential waste. We will do our best to make sure that any personal information gathered for this research study is kept private and treated in accordance with the Data Protection Act 2018 (https://www.gov.uk/government/collections/data-protection-act-2018).

Are there benefits to taking part in the study?

There will be no direct benefit to you from participating in this study. However, the information that you provide will help the Trial team and future researchers understand and improve recruitment to surgical and perioperative trials which we hope will benefit future groups of patients.

What other choices do I have if I do not take part in this study?

You are free to choose not to participate in the study. If you decide not to take part in this study, there will be no penalty to you, it will not affect your clinical care or your participation in the Trial.

What are the costs of taking part in this study? Will I be paid for taking part in this study?

There are no costs to you for taking part in this study. You will not be paid for taking part in this study.

What are my rights if I take part in this research study?

Taking part in this study is your choice. You may choose either to take part or not to take part in the study. No matter what decision you take, there will be no penalty to you in any way.

Who can answer my questions about the research study?

You can talk to the researchers about any questions or concerns you have about this study. Their contact details can be found below. If for any reason you do not wish to do this, please see the section below.

Giving consent to participate in the research study

You may keep this information sheet if you wish. Participation in this study is voluntary. You have the right to decline to participate in the study without penalty. If you do not wish to participate, you should inform the researcher when given a questionnaire, or at the beginning of the recruitment consultation/interview. If you do

For peer review only

not agree to quotes or other results arising from your participation in the study being included, even anonymously, in reports about the study, please tell the researcher.

Who is organising and funding the research study?

The study is organised by the Royal College of Anaesthetists and led by Prof SR Moonesinghe, a Consultant in Intensive Care Medicine and Anaesthesia at UCLH, and Professor of Anaesthesia and Perioperative Medicine at UCL. Prof Moonesinghe is assisted by a multidisciplinary project team consisting of anaesthetists, surgeons and patients. The research costs for the study have been supported by a grant from the National Institute for Health Research.

Who has reviewed the research study?

The study design has been reviewed by the UCL Research Ethics Committee and the Health Research Authority before any patients or staff were approached to participate.

What will happen to the results?

The results will be analysed and used to help improve processes for recruiting patients to the Trial. In addition, results will be written up for publication in scientific journals and for presentation at conferences. All information gathered will be anonymised and will not be traceable to you.

"What if there is a problem" or "What happens if something goes wrong?"

If you wish to complain, or have any concerns about any aspect of the way you have been approached or treated by members of staff you may have experienced due to your participation in the research, please contact the research team. UCL complaints mechanisms may also be available to you, or the Patient Advice and Liaison Service (PALS) at your hospital (see below).

Chief Investigator: Prof SR Moonesinghe **email:** ramani.moonesinghe@nhs.net

Researcher: Dr Duncan Wagstaff **email:Duncan.wagstaff@nhs.net**

UCL Centre for Perioperative Medicine, Charles Bell House, 43-45 Foley Street, London, W1W 7TS

For peer review only

Appendix 3: Participant Information Sheet – for recruiters

Understanding patient recruitment to a perioperative RCT. A qualitative study.

Participant Information Sheet (Recruiters)

For peer review only

Introduction

We would like to invite you to participate in this sub-study exploring recruitment to a randomised controlled trial, 'Assessing the Impact of Octreotide Infusion during Liver Transplantation' (henceforth referred to as 'the Trial'). We are interested in learning about why patients are willing (or not) to participate in the Trial. To do this, we are surveying and interviewing patients, recording recruitment consultations, and also interviewing members of the Trial team. Any contributions you make will be anonymised and collated as part of a larger group.

Why is this sub-study being done?

We hope to understand and improve processes for recruiting patients to the Trial.

What will happen if I take part in the interviews?

You may be sent an email and subsequently approached by a researcher to invite you take part in an interview. This participant information sheet will be provided in advance of you being interviewed, and with sufficient time (at least 24 hours) to be able to ask any questions you may have. Having had your questions answered satisfactorily, you will be asked to sign a consent form at the start of the interview. The interview will be conducted in private and last approximately 30-60minutes during which time we will ask you some questions about the Trial, its recruitment processes and how you think they might be improved. We will take notes of the discussion and an audio recording will also be made using a digital voice recorder. Recorded interviews will be transcribed (written up) and the tape will then be wiped clean. All information gathered will be treated as confidential by the study personnel and anonymised for analysis.

How long will the sub-study last?

The total duration of the study will be two years but your involvement will only be for your interview.

Can I stop being in the sub-study?

You can decide to stop participating at any time. After an interview has been concluded, you are free to decline consent to any future involvement in the study but historic data will not be destroyed.

What risks can I expect from being in the sub-study?

This is a very low risk study. Information you provide about your experiences and opinions will be documented, but your name will not be recorded or used in any reports of the information provided. The information obtained from these interviews will only be used by the project researchers and will be locked at our project offices. Hard copies of research data will be shredded after 10 years, and securely disposed of in confidential

waste. We will do our best to make sure that any personal information gathered for this research study is kept private and treated in accordance with the Data Protection Act 2018 (https://www.gov.uk/government/collections/data-protection-act-2018).

Are there benefits to taking part in the sub-study?

There will be no direct benefit to you from participating in this study. However, the information that you provide will help the Trial team and future researchers understand and improve recruitment to surgical and perioperative trials.

What other choices do I have if I do not take part in this sub-study?

You are free to choose not to participate in the study. If you decide not to take part in this study, there will be no penalty to you.

What are the costs of taking part in this study? Will I be paid for taking part in this sub-study?

There are no costs to you for taking part in this study. You will not be paid for taking part in this study.

What are my rights if I take part in this research sub-study?

Taking part in this study is your choice. You may choose either to take part or not to take part in the study. No matter what decision you take, there will be no penalty to you in any way.

Who can answer my questions about the sub-study?

You can talk to the researchers about any questions or concerns you have about this study. Their contact details can be found below. If for any reason you do not wish to do this, please see the section below.

Giving consent to participate in the sub-study

You may keep this information sheet if you wish. Participation in this study is voluntary. You have the right to decline to participate in the study without penalty. If you do not wish to participate, you should inform the researcher when given a questionnaire, or at the beginning of the recruitment consultation/interview. If you do not agree to quotes or other results arising from your participation in the study being included, even anonymously, in reports about the study, please tell the researcher.

Who is organising and funding the sub-study?

For peer review only

The study is by Prof SR Moonesinghe, a Consultant in Intensive Care Medicine and Anaesthesia at UCLH, and Professor of Anaesthesia and Perioperative Medicine at UCL. Prof Moonesinghe is assisted by a multidisciplinary project team consisting of anaesthetists, surgeons and patients. The research costs for the study have been supported by a grant from the National Institute for Health Research.

Who has reviewed the sub-study?

The sub-study has been reviewed by the UCL Research Ethics Committee and the Health Research Authority before any patients or staff were approached to participate.

What will happen to the results?

The results will be analysed and written up for publication in scientific journals and for presentation at conferences. All information gathered will be anonymised and will not be traceable to you.

"What if there is a problem" or "What happens if something goes wrong?"

If you wish to complain, or have any concerns about any aspect of the way you have been approached or treated by members of staff you may have experienced due to your participation in the research, please contact the research team. UCL complaints mechanisms may also be available to you.

Chief Investigator: Prof SR Moonesinghe **email:** ramani.moonesinghe@nhs.net

Researcher: Dr Duncan Wagstaff **email:Duncan.wagstaff@nhs.net**

[illegible]

For peer review only

Appendix 4: Consent Form

CONSENT FORM Participant Identification Number:

Title of Project: **Understanding Patient Recruitment to a Perioperative RCT**

Chief Investigator: **Dr Ramani Moonesinghe**

Please initial box

I confirm that I have read and understand the Participant Information Sheet (version 1.2) for the above study. I have had the opportunity to consider the information, ask questions and have had these answered satisfactorily ☐

I understand that my participation in an interview or recording of recruitment consultation is voluntary and that I am free to withdraw at any time without giving any reason, without my employment or legal rights being affected. ☐

I understand that data and quotations I provide may be used (anonymised fully) in future publications of this research. ☐

I understand that audio recordings of the interviews and/or recruitment consultations will be made and stored anonymously. The recording will be deleted once it has been written up. ☐

☐

For peer review only

I understand that, in the event of my withdrawal from the study, data I provide prior to my withdrawal will be retained (anonymised fully) for analysis and publication.

I understand that data collected during the study may be looked at by individuals from University College London (UCL) or from regulatory authorities where it is relevant to my taking part in this research. I give permission for these individuals to have access to my records. Information will be treated as strictly confidential and handled in accordance with the provisions of the General Data Protection Regulation. ☐

I agree to take part in the above study. ☐

____________________	____________	________________
Name of Participant	Date	Signature
____________________	____________	________________
Name of Person taking consent	Date	Signature

When completed: 1 for participant; 1 (original) for researcher site file

For peer review only

Appendix 5: Patient Questionnaire

Clinical Trials Questionnaire

We are interested in the reasons why patients accept or decline to take park in the clinical trial of octreotide infusion during liver transplantation. We would be grateful if you could complete and this questionnaire. It will not be shown to your doctor or any of the staff at the hospital.

	Yes	No	Not decided yet
Did you agree to take part in the trial mentioned above?			

Below are some reasons that may have influenced your decision to accept or decline to take part in this trial. Please answer each question by ticking the box that shows most clearly how you feel.

	Strongly agree	Agree to some extent	Unsure	Disagree to some extent	Strongly disagree
1) I thought the trial/study offered the best treatment available.					
2) I believed the benefits of treatment in the trial/study would out-weigh any side-effects.					
3) I was satisfied that either treatment in the trial/study would be suitable for me.					
4) I was worried that my illness would get worse unless I joined the trial/study.					
5) The idea of randomisation worried me.					

For peer review only

6) I wanted the doctor to choose my treatment rather than be randomised by computer					
7) The doctor told me what I needed to know about the trial					
8) I trusted the doctor treating me.					
9) I was given too much information to read about the trial.					
10) I was given enough information to read about the trial.					
11) I knew that I could leave the trial at any time and still be treated.					
12) I did not feel able to say no.					
13) I wanted to help with the doctors' research.					
14) I feel that others will benefit from the results of the trial.					
15) The doctor wanted me to join the trial.					
16) Others (e.g. family/friends) wanted me to join the trial.					

For peer review only

Which as the most important reason for you out of this list? (Please give number)________________

Are there any other reasons for your decision? Please list them below

Thank you very much for completing this questionnaire.

Appendix 6: Interview Topic Guide – for patients

Understanding Patient Recruitment to a Perioperative RCT.

INTERVIEW TOPIC GUIDE

1. Did you agree to take part in the study?
2. What were your reasons for agreeing/declining to take part?
3. How did you feel about the written information you received before speaking to the study team?
4. After speaking to the study team, did you understand everything that you wanted to about the trial?
5. What did you understand the potential benefits of participation might be?
6. What did you understand the potential harms of participation might be?
7. Was there any other information that you wanted to know before making your decision?
8. How did you feel about the process of randomisation?
9. Did discussions with family/friends affect your reasons for agreeing/declining to take part in the trial, and if so, in which ways?
10. Did your doctor(s) want you to take part in the trial?
11. Is there anything else that you'd like to mention?

For peer review only

Appendix 7: Interview Topic Guide – for recruiters

Understanding Patient Recruitment to a Perioperative RCT.

INTERVIEW TOPIC GUIDE - recruiters

1. Please describe your role in the study
2. How do you feel about the **written information** about the trial which is provided for participants before they are recruited? (i.e. timing, format, content)
3. How do you feel about the processes for recruiting patients?
   a. Location
   b. Timings
   c. Information sheets
   d. Consent form
4. What do you think potential participants understand the potential **benefits** of participation might be?
5. What do you think potential participants understand the potential **risks** of participation might be?
6. What are the main reasons you think that patients **agree** to take part?
7. What are the main reasons you think that patients **decline** to take part?
8. Are there any aspects of the trial which are particularly hard to explain to participants?
9. How do you think patients feel about the process of randomisation?
10. Do you think discussions with family/friends affect patients' decisions for agreeing/declining to take part in the trial, and if so, in which ways?
11. How do you think the recruitment process(es) could be improved?
12. Is there anything else that you'd like to mention?

For peer review only
